# SPECT image segmentation for estimation of tumour volume and activity concentration in ^177^Lu-DOTATATE radionuclide therapy

**DOI:** 10.1186/s13550-017-0262-7

**Published:** 2017-02-23

**Authors:** Johan Gustafsson, Anna Sundlöv, Katarina Sjögreen Gleisner

**Affiliations:** 1 Department of Medical Radiation Physics, Clinical Sciences Lund, Lund University, Lund, Sweden; 2 Department of Oncology and Pathology, Clinical Sciences Lund, Lund University, Lund, Sweden; 3 Department of Oncology, Skåne University Hospital, Lund, Sweden

**Keywords:** SPECT, Segmentation, Radionuclide therapy, Activity quantification

## Abstract

**Background:**

Dosimetry in radionuclide therapy has the potential to allow for a treatment tailored to the individual patient. One therapeutic radiopharmaceutical where patient-specific dosimetry is feasible is ^177^Lu-DOTATATE, used for the treatment of neuroendocrine tumours. The emission of gamma photons by ^177^Lu allows for imaging with SPECT (single photon emission computed tomography). One important step for dosimetry using this imaging technique is the SPECT image segmentation, which needs to be robust and accurate for the estimated quantities to be reliable. This work investigates different methods for automatic tumour delineation in ^177^Lu-DOTATATE SPECT images.

Three segmentation methods are considered: a fixed 42% threshold (FT), the Otsu method (OM) and a method based on Fourier surfaces (FS). Effects of including resolution compensation in the iterative SPECT image reconstruction are also studied. Evaluation is performed based on Monte Carlo-simulated SPECT images from 24 h and 336 h post injection (p.i.), for determination of the volume, activity concentration and dice similarity coefficient. In addition, patient data are used to investigate the correspondence of tumour volumes when delineated in SPECT or morphological CT or MR images. Patient data are also used to examine the sensitivity to the operator-dependent initialization.

**Results:**

For simulated images from 24 h p.i. reconstructed without resolution compensation, a volume and activity-concentration root-mean-square error below 15% is typically obtained for tumours above approximately 10 cm^3^ when using OM or FS, while FT performs considerably worse. When including resolution compensation, the tumour volume becomes underestimated and the activity concentration overestimated. The FS method appears to be robust to noise, as seen for the 336 h images. The differences between the tumour volumes estimated from the SPECT images and the volumes estimated from morphological images are generally larger than the discrepancies seen for the simulated data sets.

**Conclusions:**

Segmentation results are encouraging for future dosimetry of tumours with volumes above approximately 10 cm^3^. Using resolution compensation in the reconstruction may have a negative effect on volume estimation.

## Background

Radionuclide therapy (RNT) with ^177^Lu-DOTATATE is an increasingly used treatment for disseminated neuroendocrine tumours [1, 2]. Dosimetry, i.e., the determination of the absorbed dose to risk organs or tumours, is a potentially useful tool for optimization of RNT by maintaining the absorbed dose to risk organs within safe limits, while at the same time achieving as high an absorbed dose as possible to tumour to increase the likelihood of a durable anti-tumour effect [3–7]. One method for absorbed-dose estimation is by means of SPECT (single photon emission computed tomography), in which three-dimensional images of the activity distribution in a patient are obtained. In the case of ^177^Lu, SPECT-based dosimetry is possible through the emission of gamma photons at 113 keV and at 208 keV. In principle, three-dimensional images allow for estimation of absorbed dose on a voxel level, and hence characterization of the spatial distribution of absorbed dose within a structure [8]. However, SPECT suffers from a number from image degrading effects, such as noise and a poor spatial resolution, which impair the voxel-wise quantification [9], and in practice the quantity reported for clinical dosimetry is typically the mean absorbed dose to a target. The poor spatial resolution also affects the quantification of mean absorbed dose due to the spill-out effect, which is resolution-induced mispositioning of activity in the image and causes underestimation of the activity concentration in volumes with high activity.

The management of spill-out is closely connected to the strategy used for image segmentation, i.e., the definition of volumes of interest (VOIs), and the problem needs to be assessed in relation to this strategy. The magnitude of the spill-out effect depends on the size and shape of the object and the VOI placement within that object. A systematic preference to a small, centrally placed VOI will result in a different estimation of the mean concentration than if a VOI that follows the physical boundary of the object is used. A prerequisite for reliable RNT dosimetry is thus robust and reproducible SPECT segmentation methods [10], accurate in terms of the ability of properly determining the object border and the object volume, which in turn affects the accuracy of the estimated activity concentration.

The most common method for medical-image segmentation is manual delineation, a method that suffers from being both time consuming and operator-dependent [11, 12]. As an alternative, automatic or semi-automatic methods can be used and several strategies have been described [13]. For SPECT image segmentation, there are several problems caused by the image characteristics [14], and the use of automatized SPECT image segmentation for RNT dosimetry has been relatively little investigated in relation to its potential benefits and consequences for the absorbed-dose estimation. Most proposed segmentation methods for SPECT are based on thresholding, with large differences in the level of complexity ranging from a fixed threshold to adaptive or iterative methods [15–20]. Alternative techniques have also been investigated [21–23]. There is also an increasing interest for advanced segmentation methods for PET (positron emission tomography) images [24–26], which share many of the sources of image degradation with SPECT images, even if the image characteristics is typically more favourable in the PET case. One example is to use a deformable contour that is attracted to the boundary of an object in the image [27]. Deformable contours can be extended to surfaces and used in three dimensions, an example in SPECT being the adaptive surfaces described by Floreby et al. [22, 23] where Fourier descriptors were used to construct a closed surface that was adapted to an object in the image [28].

In this paper, we investigate different SPECT segmentation methods with automatic delineation for use in tumour dosimetry in ^177^Lu-DOTATATE therapy. Absorbed-dose estimation is not addressed as such, but rather the accuracy of volume and activity concentration estimation from SPECT images. An accurate estimation of these two quantities is considered essential for an accurate absorbed-dose estimation. Three different segmentation methods are considered: thresholding using a fixed threshold relative to the maximum, a threshold that is automatically determined from the image information [20, 29], and deformable surfaces based on Fourier descriptors [22, 23, 28, 30]. The segmentation is evaluated with respect to volume, dice similarity coefficient (DSC) [31] and the tumour activity concentration. As image data, Monte Carlo-simulated SPECT images [32] of anthropomorphic computer XCAT phantoms [33, 34] coupled to a pharmacokinetic model of ^177^Lu-DOTATATE [35] are used, and also a set of patient images where the tumour volumes obtained from SPECT image segmentation are compared to volumes obtained by manual delineation in diagnostic CT (computed tomography) or MR (magnetic resonance) images.

## Methods

### Segmentation methods

All of the following segmentation methods were implemented using in-house software written in the IDL programming language (Exelis Visual Information Solutions, Boulder, USA).

#### Initialization

All segmentation methods used in this study need to be initialized by manual selection of the object of interest. Practically, this was performed by manually drawing a rough VOI, encompassing the object with a margin. This initial VOI will henceforth be referred to as the initialization VOI.

#### Fixed threshold

As the technically least complicated segmentation method, a fixed threshold was used. The threshold value was set to 42% of the maximum voxel value in the initialization VOI. This segmentation method will be referred to as FT (fixed threshold). This method is included because of its simplicity and wide-spread use, with 42% being an often used value for the threshold [19].

#### Otsu method

The Otsu method (OM) automatically chooses a threshold for classifying a set of voxels as belonging to either of a background class or an object class. This method regards each voxel value as a possible threshold and tests whether a given threshold will maximize the between-class variance [20, 29]. As a segmentation method, OM is simple to implement, while at the same time it provides an automatic selection of the threshold that is adapted to the histogram of the voxel values within the initialization VOI. Hence, it constitutes a simple alternative to the FT method. Originally, OM was formulated for the grey-level histogram of an image, i.e., for an image with an equidistant quantization. Since the SPECT images in this study were coded as floating point single precision, direct application of the method would require binning of the histogram. To avoid the arbitrary division of the histogram into bins, the method was modified to an in-effect non-equidistant binning, so that every unique voxel value within the initialization VOI was regarded as a possible threshold, noting that using any threshold between two unique voxels values produced identical result in terms of voxel classification.

#### Fourier surface method

**Fig. 1:**
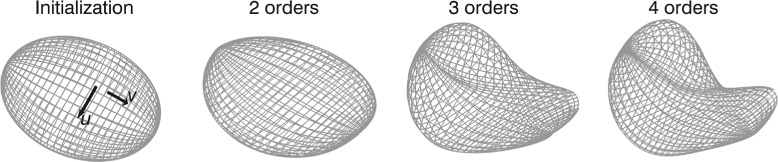
Fourier surface optimization. The Fourier surface is gradually adapted to an image object starting from an ellipsoid as initialization. In the initialization surface, the u- and v-directions are also indicated

Fourier descriptors is a method for describing curves or surfaces using Fourier series [23, 28]. The Cartesian coordinates *x*, *y* and *z* along a closed surface can be described according to

1 $$ {\begin{aligned} x\left(u,v\right)=&\;a_{x,0,0}+2a_{x,0,1}\cos(v) \\[-4pt] &+2\sum_{l=1}^{K_{2}-1}c_{x,0,l}\sin(lv) \\[-3pt] &+4\sum_{m=1}^{K_{1}-1}\sum_{l=1}^{K_{2}-1}\left[c_{\text{\textit{x,m,l}}}\cos(mu)\sin(lv)\right.\\[-3pt] &+\left.d_{\text{\textit{x,m,l}}}\sin(mu)\sin(lv)\right] \end{aligned}}  $$

2 $$ {\begin{aligned} y\left(u,v\right)=&\;a_{y,0,0}+2a_{y,0,1}\cos(v) \\[-4pt] &+2\sum_{l=1}^{K_{2}-1}c_{y,0,l}\sin(lv) \\[-3pt] &+4\sum_{m=1}^{K_{1}-1}\sum_{l=1}^{K_{2}-1}\left[c_{\text{\textit{y,m,l}}}\cos(mu)\sin(lv)\right. \\[-3pt] &+\left.d_{\text{\textit{y,m,l}}}\sin(mu)\sin(lv)\right] \end{aligned}}  $$

3 $$ {\begin{aligned} z\left(u,v\right)=&\;a_{z,0,0}+2a_{z,0,1}\cos(v) \\ &+2\sum_{l=1}^{K_{2}-1}c_{z,0,l}\sin(lv) \\ &+4\sum_{m=1}^{K_{1}-1}\sum_{l=1}^{K_{2}-1}\left[c_{\text{\textit{z,m,l}}}\cos(mu)\sin(lv)\right. \\ &+\left.d_{\text{\textit{z,m,l}}}\sin(mu)\sin(lv)\right] \end{aligned}}  $$

where *u*∈[0,2*π*) and *v*∈[0,*π*] are spatial variables along the surface, with associated wave numbers denoted by *m* and *l*, respectively. Parameters *a*, *c*, and *d* are Fourier coefficients with subscripts that indicate if they are used for describing *x*, *y* or *z* and the wave numbers *m* and *l*. The number of terms included in the Fourier series, *K*_1_ and *K*_2_ in the u- and v-directions, respectively, affect the degree of modulation that can be expressed by the surface (Fig. 1). When including higher terms in the sum, gradually more variation can be expressed. In principle, *K*_1_ and *K*_2_ can be different, but in this study they are given the same value, as there is no obvious reason to use more degrees-of-freedom for one surface direction compared to the other. A surface described using *K*_1_=*K*_2_=*K* will henceforth be referred to as a surface with *K* Fourier orders.

As the basis for segmentation of SPECT images, Fourier surfaces have the appealing property of being intrinsically resistant to noise. For SPECT, high-frequency modulation in the surface description is likely to reflect noise in the image rather than a true feature of the object considered. If only a few Fourier orders are used, only slowly varying solutions are achievable.

**Initial estimation and optimization** Segmentation using Fourier surfaces (FS segmentation) aims to determine appropriate values to the set of Fourier coefficients *a*, *c*, and *d*, for a given number of Fourier orders such that the resulting surface follows the object contour, as reflected in the SPECT image. In order to fulfil this task, an initial estimation of the coefficients of a two-order surface in the form of an ellipsoid was first performed. An optimization scheme was then applied where the number of included Fourier orders were gradually increased, starting from two and ending at four. Once convergence was reached for a given order, it was increased by 1 using an initial value of 0 for the appended coefficients.

The initial estimation of the surface consisted of determining an ellipsoid that approximated the object. The ellipsoid, as described by a centre point, three rotations, and length of three semi-axes, was estimated in a multi-step process. The initialization VOI was first thresholded using the Otsu method, following the implementation in Mortelmans et al. [20], followed by a morphological closing operation, and the centre-of-mass for the resulting binary object was used as estimate of the centre point. The directions of the semi axes were estimated by applying the Hotelling transform [36] to the binary object. The lengths of the semi axes were then determined in an optimization process so that the ellipsoid included as many of the initially-classified object-voxels as possible, while excluding voxels not classified as object.

As a measure of the edge strength along the surface, an objective function, *B*, similar to the one used by Floreby et al. [22, 23] was used

4 $$ B=\frac{\sum_{i=0}^{N_{1}-1}\sum_{j=0}^{N_{2}-1}\left[\nabla I\cdot\mathbf{n}\left(u_{i},v_{j}\right)\right]\delta\left(u_{i},v_{j},\nabla I\right)}{\left(\sum_{i=0}^{N_{1}-1}\sum_{j=0}^{N_{2}-1} \left|\mathbf{n}\left(u_{i},v_{j}\right)\right|\right)^{\alpha}},  $$

where *N*_1_ and *N*_2_ are the number of sampling points distributed along *u* and *v*, respectively, ∇*I* is the image gradient, i.e. the magnitude and direction of the change of the voxel values calculated by convolution with three 3×3×3 kernels that calculate differences in the x-, y-, and z-directions, respectively, and **n** is the surface normal. The function *δ* is used for discriminating against gradients pointing in the opposite direction compared to the contrast and current direction of the surface normal taking values of 0, 1, or -1 depending on the sign of the scalar product and the relative directions of *u* and *v*. The nominator is thus the component of the image gradient directed out- or inwards from the surface, and summed over the surface area, while the denominator is the total surface area raised to a positive scalar value *α*. The denominator, i.e. normalization to the total surface area, served the purpose of preventing *B* from increasing simply by increasing the total surface area. The reason for introducing the parameter *α* is that it provides a means to tune the penalizing effect of this normalization for the comparably low-resolution images used herein, where the image gradients are otherwise not strong enough to balance the surface from shrinking inwards. A normalization exponent of unity corresponds to a direct normalization to the total area, while a value of zero corresponds to no normalization. Fourier surfaces were adapted to the image gradient using up to four Fourier orders with surfaces realized with *N*_1_=*N*_2_=36 and a value of *α* of 0.7. For practical applications, these values may be changed by a user on a case-by-case basis, but for consistency they are kept fixed in this study. The values of *N*_1_ and *N*_2_ are the basis for in how many points Eq. (4) is evaluated. Hence, it is primarily of importance that these numbers are not set too low. The value of *α*, on the other hand, will have a more direct influence on the optimization. A value in the order 0.5 to 0.7 has in our experience produced good results when applying the segmentation method to kidneys [30].

The value of *B* was maximized using the downhill simplex method [37]. A two-stage optimization scheme was applied where one maximum was found and the optimization process was then re-initialized from the current optimum until the objective-function value did not change appreciably between consecutive re-initializations, or a predefined number of re-initializations was reached.

**Voxelization** After optimization of the Fourier surface to the object edge, a voxel mask was produced from the interior of the surface. This was done by comparing the relative directions of the surface normal and the vector pointing from a voxel coordinate to the closest point on the surface as determined in a distance minimization over the surface coordinates *u* and *v*. One potential problem in this voxelization strategy was the risk that the surface formed “folds” thereby locally changing the direction of the surface normal. This problem was addressed by imposing a criterion of the resulting voxel mask to be solid and connected by assuming that the point (*a*_*x*,0,0_,*a*_*y*,0,0_,*a*_*z*,0,0_), which determined the global surface position, was located inside the object and a voxel at the edge of the image matrix was assumed to be outside the object. Holes in the connected volume containing (*a*_*x*,0,0_,*a*_*y*,0,0_,*a*_*z*,0,0_) as well as voxels classified as part of the object but not being connected to the main volume, were removed.

### Monte Carlo-simulated SPECT images

**Fig. 2:**
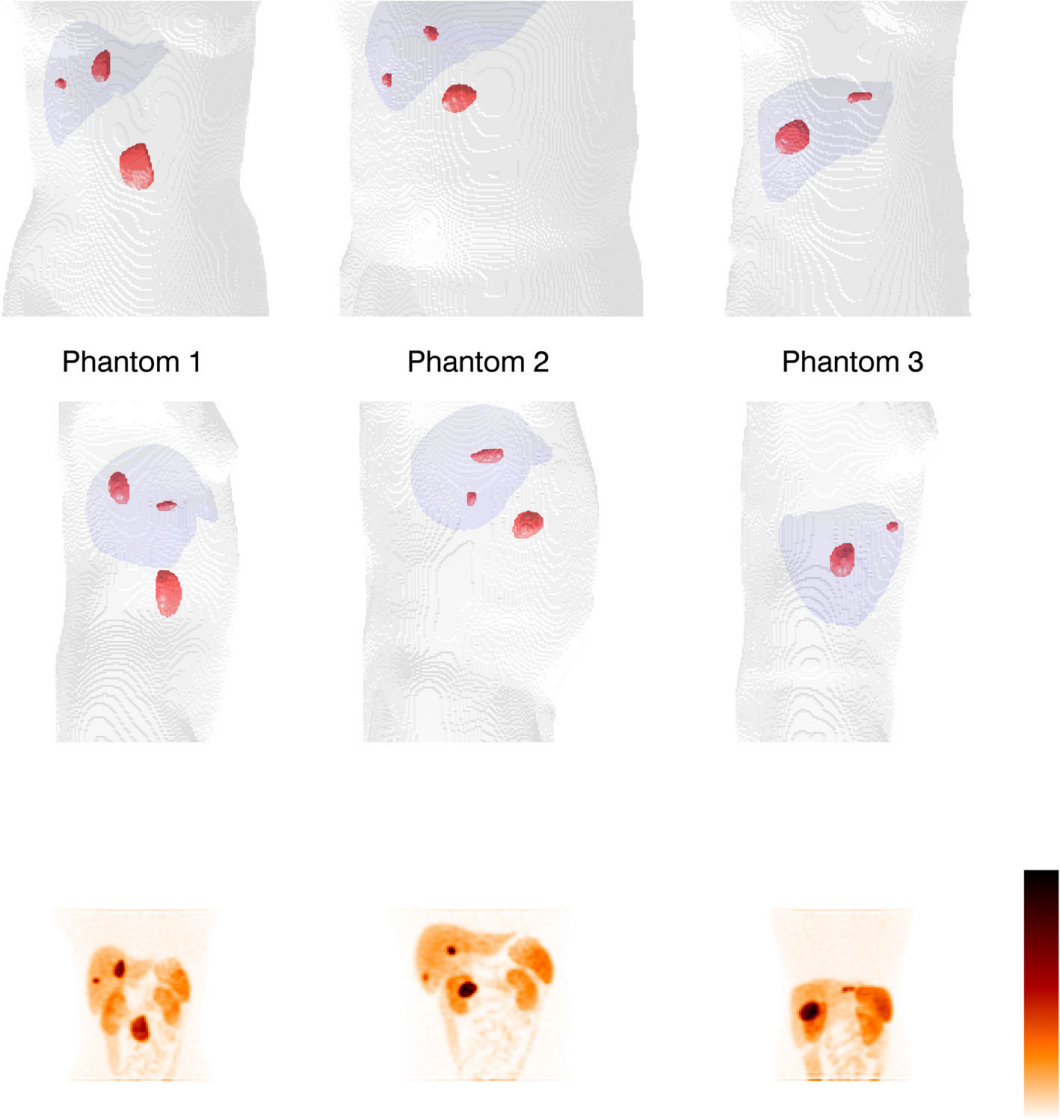
Tumour shapes and positions in the phantoms. The parts of the body outlines and the liver are included as anatomical references. The *bottom row* shows total intensity projections of the corresponding simulated SPECT images at 24 h p.i

Three phantoms from the XCAT family [33, 34] voxelized with 2.5 mm cubic voxels coupled to a pharmacokinetic model of ^177^Lu-DOTATATE [35] were used. The phantoms included two or three tumours defined by voxel masks originally obtained by delineation in patient SPECT images as described in Brolin et al. [35]. The tumour shapes and position within the phantoms are illustrated in Fig. 2. In total 8 tumours were considered with volumes ranging between 2.75 cm^3^ and 45.5 cm^3^. The imaging time-points studied were 24 h post injection (p.i.) and 336 h p.i., for which essentially noise-free SPECT projections were simulated using the SIMIND Monte Carlo program [32]. The investigation of a late imaging time-point (336 h) was motivated by the long-term tumour retention of ^177^Lu-DOTATATE which may motivate the inclusion of late measurements for improved assessment of effective half-life and thus the tumour absorbed dose [38]. Hence, an investigation of the behaviours of the segmentation methods at the count levels at a late time-point was deemed informative.

The simulated camera was equipped with a medium energy collimator, acquiring 60 projections in full rotation mode in 128×128 matrices with 4.42×4.42 m*m* ^2^ pixels, and using a 15% energy window centred at 208 keV. The simulation used an analytical collimator which means that penetration and scattering in the collimator were ignored in this study.

The output of the SIMIND program is projections giving the expected number of counts per pixel per unit of activity in the phantom and unit of time per projection. The projection pixel values images were rescaled to an injected activity of 7400 MBq at time 0 and a projection time of 45 s and using the pharmacokinetic model to account for excretion of the radiopharmaceutical from time 0 to the imaging time point. To get the noise properties of real gamma-camera projections, each pixel value was replaced by a pseudo-random number following a Poisson distribution specified by the Monte-Carlo derived expectation value. Thirty noise realizations were made, thereby giving data sets corresponding to 30 repetitions of the SPECT acquisition for each time point.

All SPECT images were reconstructed using ordered subsets expectation maximization (OS-EM) using an off-line program. Two reconstructions were performed for each SPECT study. The first employed compensation for attenuation and scatter using the effective source scatter estimation method [39] while the second used compensation for attenuation, scatter and distance-dependent resolution. The resulting image types will be referred to as AS (attenuation, scatter) and ASR (attenuation, scatter, resolution), respectively. At 24 h p.i. eight iterations and six angles per subset (AS-8-6 and ASR-8-6) were used, while at 336 h p.i. 16 iterations and 15 angles per subset (AS-16-15 and ASR-16-15) were employed. The reconstruction settings for the early time point follow what we normally use for ^177^Lu-DOTATATE at our institution [40]. For the late time point, the lower signal-to-noise ratio made it beneficial to increase the number of angles per subset.

The tumours were delineated in the SPECT images using the three segmentation methods for all noise realizations. The initialisation VOIs were identical for all noise realizations, imaging time-points, and segmentation methods, i.e. only one initialisation VOI was created for each tumour and then copied for use in all reconstructed images.

### Patient SPECT images

SPECT images from eight cycles of three different patients treated with ^177^Lu-DOTATATE were considered. Patient 1 was considered for cycles 1 and 4, patient 2 was considered for cycles 1, 2, 3, and 4, and patient 3 was considered for cycles 1 and 7. For each case a SPECT/CT image was acquired 1 day p.i. (six cases) or 4 days p.i. (two cases, patient 1 cycle 4 and patient 3 cycle 7), using 60 projections in full rotation mode with 45 s time per projection in 128×128 matrices with a pixel size of 4.0×4.0 m*m* ^2^, 20% energy window at 208 keV and a medium energy collimator. The density map used for attenuation and scatter correction was estimated from the CT study [41]. Image reconstructions were performed using the two reconstruction categories (AS and ASR) described above, with settings as for 24 h p.i. Three tumours per SPECT image (the same three tumours for every cycle) were delineated using FT, OM and FS. The initialization VOIs were identical for all segmentation methods and were drawn in the ASR images. For comparison, the same three tumours in each patient were manually delineated by an oncologist in CT or MR images originally acquired for treatment follow-up so as to estimate a morphological volume of the tumour.

### Evaluation

#### Monte Carlo-simulated SPECT images

The results from the three SPECT image segmentation methods were evaluated with respect to three aspects: volume error, DSC, and activity concentration error.

**Volume** For the three voxel phantoms a reference volume, *V*_ref,*j*_, was determined for each tumour *j* as the number of voxels defined as tumour in the Monte Carlo-simulation source maps multiplied with the source-map voxel-volume. The volumes estimated from *N*=30 SPECT noise realizations were compared with *V*_ref,*j*_. Denoting $\bar {V}_{j}=\frac {1}{N}\sum _{i=0}^{N-1}V_{i,j}$ as the mean estimated volume for tumour *j*, where *V*_*i,j*_ is the estimated volume for tumour *j* in noise realization *i*, the relative mean error $\bar {E}_{j}$ for tumour *j* was defined as

5 $$ \bar{E_{j}}=\frac{\bar{V}_{j}}{V_{\text{ref},j}}-1.  $$

The relative standard deviation (rSD) (the standard deviation normalized to the reference volume) was calculated according to

6 $$ \text{rSD}_{j}=\frac{\sqrt{\frac{1}{N-1}\sum_{i=0}^{N-1} \left(V_{i,j}-\bar{V_{j}}\right)^{2}}}{V_{\text{ref},j}}.  $$

The relative root-mean-square error (rRMSE) (the root-mean-square error normalized to the reference volume), was calculated as

7 $$ \text{rRMSE}_{j}=\frac{\sqrt{\frac{1}{N} \sum_{i=0}^{N-1}\left(V_{i,j}-V_{\text{ref},j}\right)^{2}}}{V_{\text{ref},j}}.  $$

Of these three metrics, $\bar {E_{j}}$ is a measure of the trueness of the volume estimation, rSD_*j*_ is a measure of the precision of the estimate with respect to noise, and rRMSE_*j*_ is a measure of the accuracy, where trueness, precision and accuracy are used as defined in the VIM [42].

**Dice similarity coefficients** The DSC is a measure of correspondence between two sets [31], and can in the context of image segmentation be defined as two times the volume of the overlap between two VOIs divided by the sum of the volumes of the VOIs. For the combination of phantoms and SPECT images utilized in the current work the coordinate systems of the phantoms and SPECT images differ. Hence, there is not a one-to-one correspondence between voxels possibly classified as tumours in the SPECT images and voxels defined as tumours in the phantoms. To overcome this problem, the DSC is obtained by considering the voxels as rectangular cuboids with dimensions defined by the voxel sizes and letting S_1_ being the set of points enclosed by voxels classified as tumour in the SPECT image and S_2_ being the set of points enclosed by voxels defined as tumour in the simulation source maps. The DSC is then computed as

8 $$ \text{DSC}=\frac{2\cdot\mu\left(\mathrm{S_{1}\cap S_{2}}\right)}{\mu\left(\mathrm{S_{1}}\right)+\mu\left(\mathrm{S}_{2}\right)},  $$

where *μ*(·) denotes the volume of a set.

The DSC was calculated for each tumour in all 30 noise realizations.

**Activity concentration** The tumour activity concentration was estimated from the SPECT images using the VOIs obtained from the three segmentation methods. Three combinations of VOIs and SPECT images were investigated: one with the VOIs obtained from the ASR image segmentations and applied to the ASR images (ASR/ASR), one with the VOIs obtained from the AS image segmentations and applied to the ASR images (AS/ASR), and one with the VOIs obtained from the AS images and applied to the AS images (AS/AS). The reason for including the mixed methodology (AS/ASR) was as an attempt to combine the better recovery properties of ASR images with the, as it turned out, potential advantage of improved volume estimation when using AS images for segmentation.

**Fig. 3:**
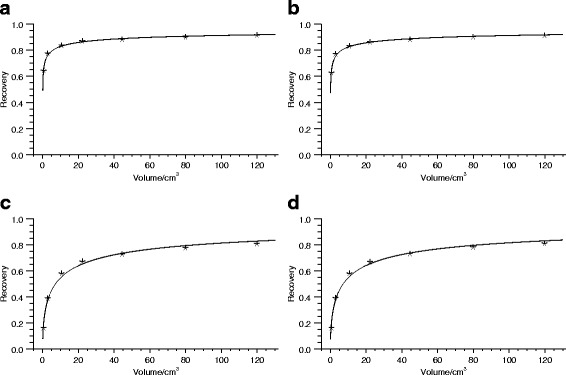
Recovery coefficients for different reconstruction schemes. The reconstruction is with attenuation and scatter correction (AS) and with or without resolution compensation (R), and different numbers of iterations and subsets. **a** ASR-8-6, **b** ASR-16-15, **c** AS-8-6, and **d** AS-16-15

In order to obtain quantitative SPECT images, the system sensitivity, i.e., the count rate per unit of activity in air, for the simulated gamma camera, was obtained by simulating a projection of a thin circular disk of ^177^Lu and summing the total signal in the projection. Partial volume effects were compensated for by recovery coefficients (RCs) obtained by Monte Carlo simulation and subsequent reconstruction of SPECT images of voxelized spheres with different volumes. The voxel spheres were located centrally in a non-radioactive, water-filled background in a voxelized elliptical cylinder with semi-axes of 20 cm and 10 cm. The voxel regions used to define the spheres in the simulations were applied as VOIs to the reconstructed SPECT images, and the ratios between the estimated activity concentration and the true activity concentration in the spheres were determined. To describe the RC as a function of voxelized-sphere volume, a relationship on the form [3, 43]

9 $$ R(V)=\frac{1}{1+\left(\frac{a}{V}\right)^{b}}  $$

was used, where *R*(*V*) denotes the RC as a function of volume *V* and *a* and *b* are two parameters fitted to data. Plots of the recovery coefficients as a function of volume are shown in Fig. 3.

The activity concentration *C* in a tumour was estimated following

10 $$ C=\frac{n}{R\left(V_{\text{tum}}\right)\cdot\varepsilon\cdot v_{\text{vox}}},  $$

where *n* is the mean count-rate per voxel in the tumour VOI for the uncalibrated SPECT image, *V*_tum_ is the estimated tumour volume, *v*_vox_ is the voxel volume and *ε* is the system sensitivity. It should be noted that the VOI is included in (10) both for *n* and for *R*.

The estimated tumour activity concentrations were compared to the reference values from the phantoms and the differences quantified as the mean error, rSD and rRMSE for each phantom as in the volume evaluation (Eqs. (5) to (7)).

#### Patient SPECT images

**Comparison of tumour delineation in SPECT and morphological images** The volumes estimated from ASR and AS SPECT images using FT, OM, and FS were compared with the VOI volume obtained from the manual delineation in the CT or MR images.

**Inter-operator variability** The inter-operator variability was evaluated for three of the patient SPECT images. Two different operators initialized the same tumours in the ASR SPECT images and the DSCs between the tumour VOIs for the two operators were calculated after application of the three segmentation methods in AS and ASR images, respectively.

## Results

### Monte Carlo-simulated images

**Fig. 4:**
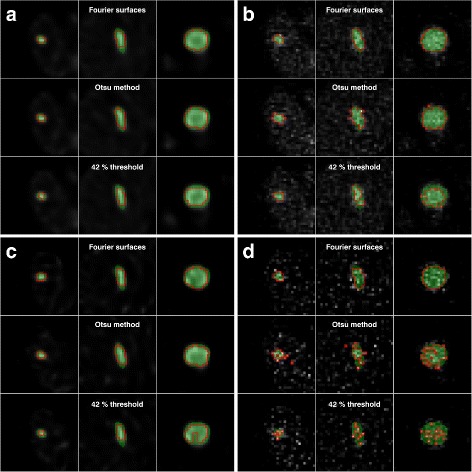
Examples of SPECT images and tumour VOIs for one phantom. **a** 24 h p.i. for ASR, **b** 24 h p.i. for AS, **c** 336 h p.i. for ASR, and **d** 336 h p.i. for AS. The green area is the VOI obtained directly from the phantom and the red contours are from the SPECT image segmentations. The SPECT images are shown in the background. The tumour volumes for each column in each sub-figure are 2.75 cm^3^ (*left*) 8.89 cm ^3^ (*middle*), and 40.0 cm^3^ (*right*)

**Fig. 5:**
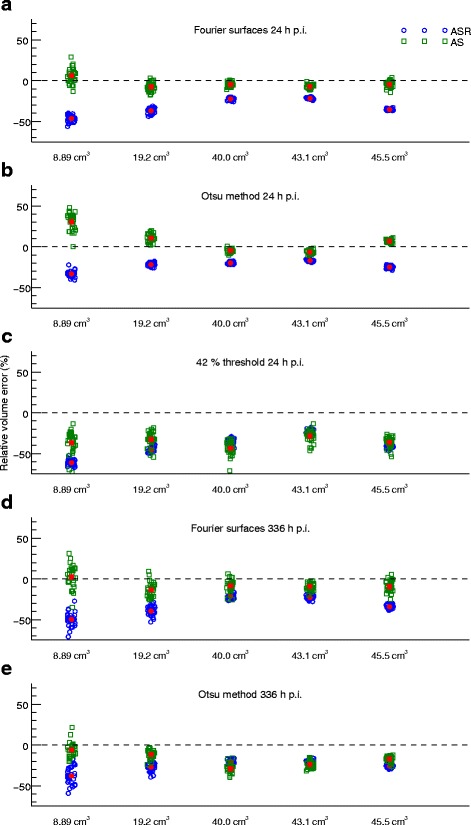
Illustration of the volume results in Tables 1 and 2. Only the five largest tumours are shown. The mean relative errors are indicated as the *red filled symbols*. The volumes along the horizontal axis are categorical only. The points have been randomly displaced in the horizontal direction to increase visibility, but this should not be interpreted as a variation in reference volume. Results are presented for ASR and AS images using **a**) FS 24 h p.i., **b**) OM 24 h p.i., **c**) FT 24 h p.i., **d**) FS 336 h p.i. and **e**) OM 336 h p.i

**Fig. 6:**
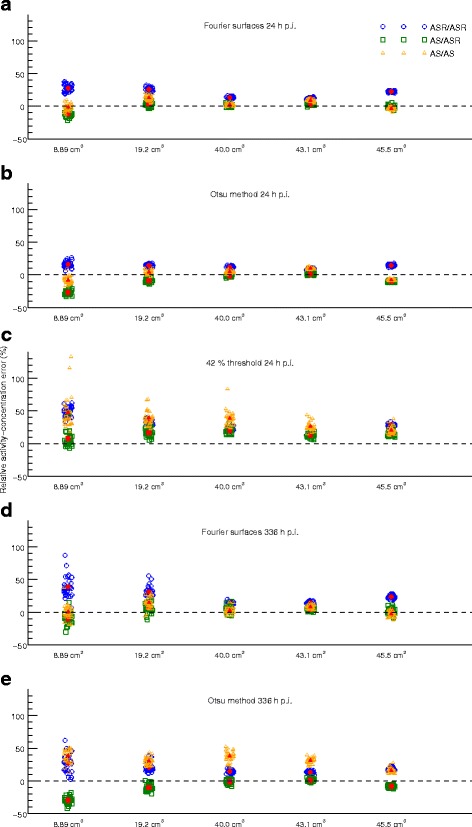
Illustration of the activity-concentration results in Tables 1 and 2. Only the five largest tumours are shown. The mean relative errors are indicated as the *red filled symbols*. The volumes along the horizontal axis are categorical only. The points have been randomly displaced in the horizontal direction to increase visibility, but this should not be interpreted as a variation in volume. Results are presented for ASR/ASR, AS/ASR and AS/AS using **a**) FS 24 h p.i., **b**) OM 24 h p.i., **c**) FT 24 h p.i., **d**) FS 336 h p.i. and **e**) OM 336 h p.i

Examples of SPECT images and tumour VOIs for one of the phantoms for the two time-points and three segmentation techniques are given in Fig. 4. The different sizes and shapes of the tumours and the larger voxel-to-voxel value variation in AS images compared to ASR images can be noted. The volume error, DSC and activity-concentration error for the different tumours in the anthropomorphic phantoms at 24 h p.i. are given in Table 1. The corresponding results at 336 h p.i. are given in Table 2. Graphical representations of the results focusing on the five largest tumours are shown in Fig. 5 (volume) and Fig. 6 (activity concentration). For the 24 h time-point, the results for all three segmentation methods are given, while for the 336 h time-point the results for FT are omitted since the quality of the VOIs was so poor that evaluation in terms of volume or activity concentration was not deemed meaningful. This can be seen in Fig. 4 where the segmentation results and VOI errors for FT at 336 h p.i. are included, and are seen to produce spurious, non-connected, voxel islands.

**Table 1:** Results for Monte Carlo-simulated SPECT images at 24 h p.i. in terms of volume error, DSC, and activity-concentration error

Method	Volume/cm^3^	Volume error (%)		DSC		Activity concentration error (%)		
		ASR	AS	ASR	AS	ASR/ASR	AS/ASR	AS/AS
FT	2.75	−57±9 (57)	−3±22 (22)	0.58 (0.46, 0.65)	0.69 (0.62, 0.73)	53±16 (55)	−3±12 (12)	50±26 (56)
	3.39	−62±4 (62)	−4±17 (17)	0.54 (0.49, 0.58)	0.70 (0.65, 0.75)	61±9 (62)	−3±11 (11)	39±18 (43)
	5.30	−50±5 (50)	−1±18 (17)	0.64 (0.58, 0.70)	0.71 (0.66, 0.74)	37±7 (38)	−8±9 (12)	18±18 (25)
	8.89	−61±4 (61)	−37±13 (39)	0.56 (0.51, 0.59)	0.67 (0.56, 0.70)	48±7 (49)	8±12 (14)	47±25 (53)
	19.2	−45±3 (45)	−33±9 (34)	0.71 (0.67, 0.74)	0.75 (0.68, 0.80)	34±3 (34)	16±6 (17)	39±11 (41)
	40.0	−35±3 (35)	−44±8 (44)	0.79 (0.75, 0.81)	0.70 (0.63, 0.77)	23±2 (23)	20±4 (20)	39±12 (40)
	43.1	−23±2 (23)	−28±7 (29)	0.86 (0.84, 0.87)	0.81 (0.72, 0.84)	13±1 (13)	13±4 (13)	26±7 (27)
	45.5	−41±2 (41)	−36±7 (36)	0.74 (0.72, 0.75)	0.75 (0.68, 0.78)	28±2 (28)	15±4 (16)	21±6 (22)
OM	2.75	−13±9 (16)	107±29 (110)	0.77 (0.73, 0.78)	0.62 (0.56, 0.67)	9±7 (11)	−39±6 (39)	−13±8 (15)
	3.39	−33±3 (33)	64±19 (67)	0.72 (0.70, 0.74)	0.67 (0.63, 0.71)	23±5 (23)	−30±6 (31)	−4±9 (9)
	5.30	−30±5 (30)	69±13 (70)	0.75 (0.73, 0.77)	0.68 (0.65, 0.69)	17±5 (18)	−33±3 (33)	−17±5 (18)
	8.89	−33±4 (33)	31±11 (32)	0.76 (0.73, 0.78)	0.71 (0.69, 0.73)	16±5 (17)	−27±4 (27)	−8±6 (9)
	19.2	−22±2 (22)	11±5 (12)	0.84 (0.83, 0.85)	0.83 (0.82, 0.84)	14±2 (14)	−9±3 (9)	4±3 (5)
	40.0	−20±1 (20)	−5±2 (5)	0.86 (0.86, 0.87)	0.86 (0.85, 0.88)	12±1 (12)	−2±1 (2)	5±2 (5)
	43.1	−17±1 (17)	−7±2 (7)	0.88 (0.88, 0.89)	0.88 (0.87, 0.88)	10±1 (10)	1±1 (2)	10±1 (10)
	45.5	−25±2 (25)	7±2 (7)	0.84 (0.83, 0.85)	0.85 (0.85, 0.86)	14±1 (15)	−10±1 (10)	−7±1 (7)
FS	2.75	−17±7 (18)	25±14 (29)	0.75 (0.73, 0.77)	0.77 (0.74, 0.78)	10±6 (12)	−12±7 (14)	19±10 (21)
	3.39	−32±5 (32)	42±10 (43)	0.69 (0.67, 0.72)	0.74 (0.71, 0.75)	20±6 (21)	−21±4 (22)	2±5 (6)
	5.30	−22±5 (23)	45±9 (46)	0.75 (0.74, 0.77)	0.75 (0.73, 0.76)	9±5 (11)	−24±3 (24)	−12±4 (12)
	8.89	−46±4 (47)	6±9 (11)	0.67 (0.63, 0.71)	0.80 (0.78, 0.82)	27±6 (28)	−12±4 (13)	−1±5 (5)
	19.2	−37±3 (37)	−8±5 (9)	0.77 (0.73, 0.79)	0.86 (0.85, 0.87)	26±3 (26)	3±4 (5)	13±4 (14)
	40.0	−22±1 (22)	−5±3 (6)	0.85 (0.84, 0.86)	0.90 (0.89, 0.91)	13±1 (13)	1±2 (2)	3±2 (3)
	43.1	−22±1 (22)	−7±2 (7)	0.86 (0.86, 0.87)	0.90 (0.90, 0.91)	12±1 (12)	3±2 (3)	9±2 (9)
	45.5	−35±1 (35)	−5±4 (6)	0.78 (0.77, 0.79)	0.88 (0.87, 0.89)	22±1 (22)	−1±2 (3)	−3±2 (4)

Results for volume and activity concentration are given as $\bar {E_{j}}\pm \text {rSD}_{j}\,\left (\text {rRMSE}_{j}\right)$. For the DSC the 50th (10th, 90th) percentiles are given. Results are given for segmentation of SPECT images reconstructed with AS and ASR. For activity concentration, results are presented for combinations of ASR and AS, for example AS/ASR where the first (AS) specifies the SPECT image used for tumour delineation while the second (ASR) refers to the SPECT images on which the resulting VOIs were applied

**Table 2:** Results for Monte Carlo-simulated SPECT images at 336 h p.i. in terms of volume error, DSC and activity-concentration error

Method	Volume/cm^3^	Volume error (%)		DSC		Activity concentration error (%)		
		ASR	AS	ASR	AS	ASR/ASR	AS/ASR	AS/AS
OM	2.75	−23±15 (27)	119±43 (126)	0.73 (0.65, 0.76)	0.44 (0.40, 0.50)	24±21 (31)	−52±10 (53)	−5±22 (22)
	3.39	−38±11 (39)	54±23 (59)	0.69 (0.59, 0.74)	0.56 (0.49, 0.60)	36±19 (41)	−36±8 (36)	9±14 (16)
	5.30	−33±7 (34)	37±17 (40)	0.72 (0.70, 0.76)	0.57 (0.53, 0.61)	25±10 (27)	−35±5 (35)	4±11 (11)
	8.89	−38±10 (39)	−6±9 (10)	0.73 (0.64, 0.77)	0.52 (0.48, 0.57)	27±15 (31)	−29±6 (30)	39±10 (40)
	19.2	−27±5 (27)	−12±5 (13)	0.81 (0.78, 0.84)	0.67 (0.65, 0.70)	21±7 (22)	−10±5 (11)	30±6 (31)
	40.0	−20±2 (20)	−29±6 (30)	0.86 (0.84, 0.87)	0.68 (0.66, 0.72)	15±2 (15)	−1±3 (3)	38±7 (39)
	43.1	−20±2 (20)	−24±4 (24)	0.87 (0.86, 0.88)	0.74 (0.72, 0.77)	13±2 (13)	1±2 (3)	32±4 (32)
	45.5	−26±2 (26)	−17±3 (17)	0.83 (0.81, 0.84)	0.71 (0.69, 0.73)	18±2 (18)	−8±2 (8)	16±4 (17)
FS	2.75	−20±6 (21)	35±25 (43)	0.74 (0.71, 0.76)	0.73 (0.68, 0.76)	16±10 (19)	−16±13 (21)	8±19 (20)
	3.39	−31±7 (32)	25±26 (36)	0.69 (0.65, 0.74)	0.72 (0.67, 0.75)	24±10 (26)	−14±13 (19)	8±16 (18)
	5.30	−25±6 (25)	33±17 (37)	0.74 (0.72, 0.76)	0.73 (0.70, 0.75)	14±6 (15)	−21±7 (22)	−10±8 (13)
	8.89	−50±9 (50)	2±14 (14)	0.65 (0.57, 0.73)	0.77 (0.71, 0.81)	39±16 (42)	−10±9 (13)	1±11 (11)
	19.2	−39±6 (40)	−13±9 (16)	0.74 (0.68, 0.80)	0.84 (0.80, 0.86)	31±8 (32)	7±7 (10)	15±8 (17)
	40.0	−21±2 (21)	−8±7 (11)	0.86 (0.84, 0.87)	0.88 (0.82, 0.89)	14±2 (14)	4±5 (6)	3±5 (6)
	43.1	−23±2 (23)	−9±4 (10)	0.86 (0.84, 0.87)	0.89 (0.88, 0.90)	14±2 (14)	4±3 (5)	9±3 (9)
	45.5	−34±2 (34)	−9±7 (11)	0.78 (0.76, 0.81)	0.86 (0.84, 0.87)	24±3 (24)	2±5 (5)	−3±4 (5)

Results for volume and activity concentration are given as $\bar {E_{j}}\pm \text {rSD}_{j}\,\left (\text {rRMSE}_{j}\right)$. For the DSC the 50th (10th, 90th) percentiles are given. Results are given for image segmentation on SPECT images reconstructed with AS and ASR. For activity concentration, results are presented for combinations of ASR and AS, for example AS/ASR where the first (AS) specifies the SPECT image used for tumour delineation while the second (ASR) refers to the SPECT images on which the resulting VOIs were applied. Results of FT have been omitted due to its poor performance causing the results not being deemed meaningful

Segmentation of SPECT images reconstructed using ASR generally produces VOIs that are smaller than the physical extension of the object. This is seen in Fig. 4 where the red contours, marking the contours of the VOIs obtained from the segmentation of the SPECT images, are typically more contracted than the green region, marking the tumours as defined in the phantom. In Tables 1 and 2, the tumour volume is thus systematically underestimated for ASR images, irrespective of the segmentation method used. Even if the relative standard deviation is low for the volume estimation from these images, this systematic deviation yields a relatively high volume-rRMSE. The volume underestimation when using ASR images for segmentation consequently produces an overestimation of the activity concentration, since a contracted VOI in general gives a different (higher) recovery than a VOI that follows the object boundary. Furthermore, a volume underestimation results in a lower RC being used, which also add to the overestimation of the activity concentration. For SPECT images at 24 h p.i. when instead using FS or OM for delineation in AS images, the estimated volume for tumours above approximately 10 cm^3^ is relatively accurate, with volume-rRMSEs of approximately 10%.

For AS/AS the activity-concentration rRMSE for FT is 22% to 56%, for OM 5% to 18%, and for FS it is 3% to 21%. The corresponding results for AS/ASR are 11% to 20%, 2% to 39%, and 2% to 24%, for FT, OM, and FS, respectively. There are tendencies for direction of the activity-concentration error (i.e., underestimation versus overestimation) to depend on volume, with the activity concentration in small volumes being underestimated. For FT the best result are obtained for AS/ASR, with activity concentration rRMSEs within 20%. For FS and OM, the activity concentration rRMSEs are typically within 10% for the larger tumours using AS images for delineation and are for AS/AS within 21% for all volumes. On the whole, the DSC is highest for the FS method applied to AS images, although the differences between OM and FS are relatively modest. For FT the DSC is generally lower. At the later time-point (336 h p.i. Table 2), the main difference compared to the 24 h results, apart from the failure of FT to produce meaningful VOIs, is a poorer performance for OM, especially in terms of DSC. Also in terms of volume and activity concentration, the FS method tends to yield better results than OM. For the four largest tumours, the volume rRMSE is between 10% and 16% when applying FS to AS images and the activity concentration rRMSE is within 10% except for one case. Irrespective of tumour volume, the activity-concentration rRMSE is between 5% and 22% for these cases.

### Clinical images

#### Comparison of tumour delineation in SPECT and morphological images

**Fig. 7:**
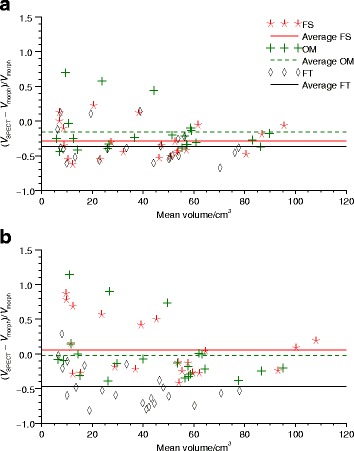
Volume differences between manual delineation in morphological images and SPECT image segmentation. The three methods FT, OM, and FS are used. SPECT images were reconstructed with **a** ASR and **b** AS. Differences are expressed as the volume differences divided by the volume derived from morphological images and are shown as function of the average volumes derived from SPECT and morphological images

Comparison of the tumour volumes estimated using SPECT and manual delineation in CT or MR images are shown in Fig. 7, and is presented in terms of the ratio of the volume difference and the volume estimated in morphological images. The average ratio for each method is given to indicate trends for over- or underestimation. In total, 24 tumours were considered, with two tumours excluded from the analysis since they were not completely within the field-of-view of the morphological images. For AS, on average the volumes obtained using FS and OM are approximately the same as the volume from manual delineation in the morphological images, while for ASR the SPECT volumes are generally smaller than their manually delineated MR or CT counterparts. For AS the average ratios are 6%, −2%, and −47% for FS, OM, and FT, respectively. For ASR the corresponding values are −29%, −16%, and −37%. However, there is a considerable spread around these averages with typically larger differences (in a relative meaning) for smaller volumes.

#### Inter-operator variability

The DSC between the resulting tumour VOIs for the two operators are presented in Table 3. When using ASR the DSCs are on average 0.93, 0.86, and 0.91 for FS, OM and FT, respectively, while when using AS the corresponding results are 0.93, 0.84, and 0.92. The poor agreement for some tumours can be noted, which can partly be explained by considerable geometric differences in the identification of the tumour by the two operators, for example in tumour 2, causing the DSC to be low for the threshold-based methods.

**Table 3:** DSC between VOIs resulting from initialization by two different operators in patient SPECT images

Tumour number	Volume^a^/cm^3^	ASR			AS		
		FS	OM	FT	FS	OM	FT
1	63	0.96	0.85	0.96	0.95	0.79	0.97
2	14	0.96	0.55	0.38	0.93	0.59	0.53
3	43	0.99	0.99	1.0	0.98	0.97	1.0
4	42	0.98	0.98	1.0	0.96	0.96	0.98
5	29	0.67	0.85	0.87	0.65	0.85	0.88
6	10	0.97	0.72	0.99	0.97	0.61	0.94
7	30	0.99	0.93	1.0	0.98	0.88	1.0
8	49	0.92	0.94	0.96	0.97	0.93	0.96
9	81	0.97	0.98	0.99	0.97	0.97	0.99

^a^Calculated as the average for FS and OM in AS images for the two operators

## Discussion

In this study, three semi-automatic segmentation methods for tumour delineation have been investigated in the context of ^177^Lu-DOTATATE therapy SPECT images, using both Monte Carlo-simulated images of anthropomorphic phantoms and patient images as test material. The methods investigated are a 42% threshold, the Otsu method, and a deformable surface method based on Fourier descriptors. Investigated aspects are the volume accuracy, the agreement between regions using DSC and the activity-concentration accuracy.

When comparing the three segmentation methods, FS and OM tend to produce approximately equivalent results for SPECT images acquired at 24 h p.i., while Fourier surfaces perform better for images acquired at 336 h p.i. This difference is particularly pronounced for the DSC results, where some of the VOIs produced by OM would hardly be considered acceptable due to their excessive jaggedness, as illustrated in Fig. 4. The FT method performs worse than the other two methods for most cases in the simulated data sets. Generally, the performance of segmentation methods heavily relies on the particular image characteristics, for instance the degree of detail exhibited by the object under consideration, the noise properties of the image, and the spatial resolution. Extrapolation of the detailed results from this evaluation to other situations in terms of for example reconstruction methods and quantification methodology can thus not be made unreservedly. Rather, the present study should be seen as a demonstration of the importance of image segmentation for an accurate activity quantification. Sometimes a simple methodology like FT is not sufficient in order for the results to be reliable, while there are other methods that are capable of produce an accurate estimate of the volume and activity quantification for a wider range of situations, for example with respect to the image noise characteristics. Optimization of the settings in the tomographic reconstruction, possible post-filtration of the reconstructed image and tuning of the parameters associated with the different segmentation methods is possible, and such optimization is often desirable for a given application. However, the less dependent a method is on such tuning, the more robust and less subjective the result. The ability of the FS method to give relatively accurate delineations also for the SPECT images corresponding to 336 h p.i., would be considered a considerable advantage compared to FT and OM. Hence, if the intention is to follow the tumour activity-concentration over long period of times, or if the reconstructed image for some other reason have unusually high noise levels, such a method may be worth pursuing. One advantage of the Otsu method is its relative simplicity and it can also be noted that in the FS method, OM is involved in the creation of the initial ellipsoid.

In general, the tumour volumes estimated in ASR images have a negative bias. We have no complete understanding of this phenomenon, but one hypothesis is that it is linked to the high values often observed close to the object edge when including resolution modelling in the reconstruction. For the threshold-based segmentation methods, if these artefacts increase the maximum value in the VOI the absolute threshold value will also increase, implying a lower estimated volume. As a segmentation method, FS is more complicated and as a consequence it is also harder to explain the behaviour with respect to ASR images. However, since the reconstruction artefacts previously mentioned cause an accumulation of signal in the object at its borders, they may also have an impact on the behaviour of a segmentation algorithm based on a surface being attracted to that edge. When reconstructing the SPECT images without resolution compensation, any potential bias in the volume estimation is less evident, and on the whole the rRMSE is lower compared to the ASR images, provided that the tumours are large enough. The size limit below which the volume estimation cannot be trusted (approximately 10 cm^3^ in this study) is likely dependent on several factors linked to the image characteristics, but the major reason is probably the image spatial resolution. Hence, in case of an improvement in this property, this size limit would decrease. It can be noted that since the volume will roughly scale as the cube of the length of the considered object, even a relatively small improvement in resolution may have an impact on what tumour volumes may be accurately estimated.

The accuracy of the quantification of activity concentration depends on a number of factors in addition to the segmentation of the image. The foundation for image-based activity quantification in nuclear medicine is the tomographic reconstruction and the correction methods included in that algorithm. If proper compensation for the major physical effects, in particular attenuation and scatter in the patient, is not performed, there is less hope of a reliable quantification. Accordingly, in terms of activity concentration, the numeric results achieved in this study are not necessarily generalizable between reconstruction programs. On the other hand, in terms of the segmentation itself and the resulting estimated volumes, the method used for, e.g., scatter compensation may be of less importance.

Concerning the realism of the simulated images, an image-degrading effect that has not been considered is patient movement, which would add further blurring to the already poor spatial resolution. However, if motion blurring were the main reason for poor volume agreement in patient images, we believe this would be manifested as a volume overestimation rather than an underestimation. So, while motion could be a relevant explanation for poor correspondence when the SPECT-derived volume is considerably larger than the morphological volume, we believe it is less likely a cause for underestimation.

Another limitation is that the tumours introduced in the phantoms, even if not simple spheres, are of relatively round shapes. This could be relevant for the segmentation evaluation, as investigated by Berthon et al. [44] for PET image segmentation. Furthermore, the phantom tumour burden is relatively low compared to most patients. This aspect could be of importance since one difficulty is to separate nearby high-activity objects from each other. Thus, particularly difficult tumour shapes and background distributions, also including the potential for non-homogeneous activity in the tumours themselves, are not fully mimicked by the simulated data.

Despite the aforementioned limitations of the simulated images, they also have a number of advantages. In particular, the anatomy and activity distribution, through the use of a pharmacokinetic model coupled to the phantoms [35], are designed to be realistic substitutes for patient SPECT images, while at the same time establishing a ground truth with respect to volume and activity concentration. The simulations allow for calculation of the DSC for the tumours. It can here be noted that the comparisons are not performed between volumes with the same voxel sizes, and rather than counting the number of voxels contained in both sets the overlapping volume is considered. Hence, part of the mismatch between the volume defined as tumour in the phantoms and the volume classified as tumour in the SPECT images is due to the relation between the phantom-image grid and the SPECT-image grid, and the voxel volume becomes a factor that affects the calculated DSCs.

When considering results in Fig. 7 of patient tumour volumes, where VOIs delineated manually in morphological images and VOIs obtained from ^177^Lu-DOTATATE therapy SPECT images are compared, the deviations for individual tumours are larger than deviations typically obtained for the simulated data sets, where SPECT-derived volumes are compared to true volumes. Comparison with morphological images have previously been used to evaluate the performance of automatic segmentation methods in for example FDG and ^124^I PET images [45]. A natural question to ask is whether the different results between simulated and patient SPECT images reflect limitations in the realism of the simulated images, i.e. if the simulated images are relevant as test material, whether differences are result of inaccuracy in the manual delineation in morphological images, or if discrepancies in patients reflect a true difference between the morphological tumour extension and the volume accumulating ^177^Lu-DOTATATE. It can also be noted that the acquisition parameters were slightly different for the simulated and patients cases. These different acquisition parameters reflect a change of gamma camera at our institution, where the simulated settings are for the new system while the patient material used in this study was acquired using the old system. These differences are likely too small to cause any substantial difference in the performance of the segmentation methods.

If considering the limitations of the clinical images it can be noted that manual segmentation in itself is not error-free. Hence, the differences seen in Fig. 7 are to some degree a combination of inaccuracies of both methods. As a consequence, it is not obvious that the morphological volume should be in the denominator in Fig. 7, and normalizing to the SPECT volume would in principle be an equally valid choice. For this reason, the individual points and average ratios indicated in the figure should be interpreted as an indication only of systematic differences and dispersion in estimated volumes. There is also the possibility of the tumour growing or shrinking (as an effect of treatment) in the time that lapses between the radiological evaluation (CT/MR) and the SPECT-imaging time-point. Neuroendocrine tumours are, however, slow-growing in nature, thereby reducing this risk. In these particular patients we also have the RECIST-measurements confirming the stable nature of the tumours all through the treatment period with the possible exception of the first imaging time-point for patient 2, whose tumour progressed moderately between the baseline CT and the first SPECT, although in RECIST-terms the disease was still stable (less than 20% increase in tumour diameter).

**Fig. 8:**
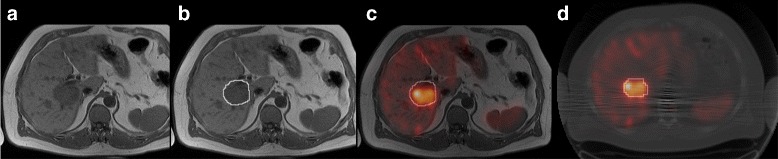
Difference for a tumour between a morphological image and SPECT. **a** MR image with **b** manually delineated morphological tumour volume. **c** Fusion of MR and co-registered SPECT images with morphological volume delineated. **d** SPECT/CT image with delineated SPECT volume using Fourier surfaces

A perhaps more interesting possible cause for the volume disagreement in patient images, rather than potential methodological limitations, is if the morphological images and SPECT images do not reflect the same underlying volume. Such a discrepancy could be relevant in terms of understanding the therapeutic effect. A volume definition solely based on activity uptake as depicted in the SPECT images will not consider tumour tissue that is either necrotic or de-differentiated and thus without expression of the sst_2_ receptor necessary for uptake of the radiopharmaceutical. An illustration of the differences between the VOIs obtained from delineation in morphological images and SPECT images for one of the patients is shown in Fig. 8. In Fig. 8c, where the manually delineated morphological volume is shown on co-registered MR and SPECT images, there appears to be a difference in the tumour volume visualized between the two modalities, with a smaller volume for SPECT. The co-registration has not been used in the evaluation and is only given here for illustrative purposes. The possible volume differences between morphological images and SPECT images and their clinical implications would be of interest for further studies. A prerequisite in such investigations is a good understanding of the segmentation method used and the uncertainties of the estimated volumes.

The small investigation on initialization dependence highlights some strengths and shortcomings of the three segmentations methods in clinical images. In particular there are some results in Table 3 which indicate a large difference between VOIs resulting from different initializations. Tumour 5 was in close proximity to another tumour (tumour 6) leading to poor local contrast, thus making it possible for the optimization to find an optimum when both tumours were included in the VOI as well as when only one of them was included. For tumour 2 there was disagreement between the two operators whether a high-activity region represented one single or two separate tumours, leading to markedly different initialization VOIs. Hence, the threshold-based methods resulted in markedly different VOIs, while the FS method encompassed approximately the same region in both cases since the result of the optimization process is not constrained by the initialization VOI. Tumour 6 was in close proximity with tumour 5 and was also located at the edge of the liver, leading to a non-uniform background that could explain the disagreement for OM.

When constructing the simulated RC curves, the simulation-defined volumes and VOIs have been used. This is an obvious idealization to a real measurement procedure where the sphere positions and volumes can never be known exactly and has to estimated using, e.g., the CT. Another possible solution would be to use the SPECT image as the basis also for this segmentation, using the same method as will later be used for the patient images. Such a strategy may have the advantage to partially cancel errors in the activity-concentration estimation, if it is assumed that the behaviour of the method is the same for spheres in a phantom as in for tumours in patients, and could thus be interesting as a way to reduce the uncertainty of the activity concentration estimation. Such a strategy might have the potential to improve an existing method, but we would at the same time argue that a method that follows the outline of an object as closely as possible is to be preferred rather than a method that relies on such potential cancellation. The use of recovery coefficients as a method to compensate for spill-out relies on the assumption that the conditions under which the coefficients are derived are similar to the patient situation. The situation in patients may be more diverse than what has been covered in the XCAT phantoms. In principle, any deviation in terms of contrast and tumour shape may affect recovery, which in such cases would call for more aspects than volume to be investigated [46, 47]. However, when applied to the simulated SPECT images used in this work the result is relatively successful even for such a simple correction model.

The typical quantity considered in RNT dosimetry is not the mean activity concentration in a volume at a single time-point but rather the mean absorbed dose to that volume. The current study is believed to be of importance for RNT tumour dosimetry where one of the critical steps is to accurately determine the activity concentration at different time-points p.i. as this is the basis for a correct absorbed-dose estimation. For the radionuclide ^177^Lu, the absorbed-dose rate is essentially proportional to the activity concentration. The resulting error in the absorbed dose will also depend on factors such as the choice of fitting function, the fitting method, and the error correlation between time points.

## Conclusions

The accuracy of semi-automatic segmentation is affected by a number of factors coupled to the characteristics of SPECT images. In Monte Carlo-simulated images a good tumour volume and activity concentration accuracy is obtained for two segmentation methods (Otsu method and Fourier surface method) when images are reconstructed with attenuation and scatter compensation, while if including resolution compensation underestimated volumes and overestimated activity concentrations are obtained. Using an adaptive surface based on Fourier descriptors has advantages if the image noise levels are high, for example if the tumour activity retention is to be followed for a long period of time. For images with less noise, for example early imaging time-points in the course of radionuclide therapy dosimetry, an adaptive thresholding algorithm like the Otsu method yields approximately equivalent results as the Fourier surface method. The results are encouraging for application in tumour dosimetry, although challenges can be foreseen due to the diversity of patient images. Further studies of the differences in tumour extension when visualized in functional SPECT and in morphological CT or MR images are of interest.

## Abbreviations

AS: Attenuation, scatter
ASR: Attenuation, scatter, resolution
CT: Computed tomography
DSC: Dice similarity coefficient
FS: Fourier surface
FT: Fixed threshold
MR: Magnetic resonance
OM: Otsu method
OS-EM: Ordered subsets expectation maximization
rRMSE: Relative root-mean-square error
rSD: Relative standard deviation
SPECT: Single photon emission computed tomography
VOI: Volume of interest

## References

[1] Kwekkeboom DJ, Teunissen JJ, Bakker WH, Kooij PP, de Herder WW, Feelders RA, van Eijck CH, Esser JP, Kam BL, Krenning EP (2005). Radiolabeled somatostatin analog [ ^177^Lu-DOTA ^0^,Tyr ^3^]octreotate in patients with endocrine gastroenteropancreatic tumours. J Clin Oncol.

[2] Bodei L, Kidd M, Paganelli G, Grana CM, Drozdov I, Cremonesi M, Lepensky C, Kwekkeboom DJ, Baum RP, Krenning EP, Modlin IM (2015). Long-term tolerability of PRRT in 807 patients with neuroendocrine tumours: the value and limitations of clinical factors. Eur J Nucl Med Mol Imaging.

[3] Ilan E, Sandström M, Wassberg C, Sundin A, Garske-Román U, Eriksson B, Granberg D, Lubberink M (2015). Dose response of pancreatic neuroendocrine tumours treated with peptide receptor radionuclide therapy using ^177^Lu-DOTATATE. J Nucl Med.

[4] Strigari L, Konijnenberg M, Chiesa C, Bardies M, Du Y, Sjögreen Gleisner K, Lassmann M, Flux G (2014). The evidence base for the use of internal dosimetry in the clinical practice of molecular radiotherapy. Eur J Nucl Med Mol Imaging.

[5] Bodei L, Cremonesi M, Ferrari M, Pacifici M, Grana CM, Bartolomei M, Baio SM, Sansovini M, Paganelli G (2008). Long-term evaluation of renal toxicity after peptide receptor radionuclide therapy with ^90^Y-DOTATOC and ^177^Lu-DOTATATE: the role of associated risk factors. Eur J Nucl Med Mol Imaging.

[6] Barone R, Borson-Chazot F, Valkema R, Walrand S, Chauvin F, Gogou L, Kvols LK, Krenning EP, Jamar F, Pauwels S (2005). Patient-specific dosimetry in predicting renal toxicity with ^90^Y-DOTATOC: Relevance of kidney volume and dose rate in finding a dose-effect relationship. J Nucl Med.

[7] Pauwels S, Barone R, Walrand S, Borson-Chazot F, Valkema R, Kvols LK, Krenning EP, Jamar F (2005). Practical dosimetry of peptide receptor radionuclide therapy with ^90^Y-labeled somatostatin analogs. J Nucl Med.

[8] Bolch WE, Bouchet LG, Robertson JS, Wessels BW, Siegel JA, Howell RW, Erdi AK, Aydogan B, Costes S, Watson EE (1999). MIRD pamphlet no. 17: The dosimetry of nonuniform activity distributions—radionuclide S values at the voxel level. J Nucl Med.

[9] Ljungberg M, Sjögreen-Gleisner K (2011). The accuracy of absorbed dose estimates in tumours determined by quantitative SPECT: A Monte Carlo study. Acta Oncol.

[10] Sandström M, Ilan E, Karlberg A, Johansson S, Freedman N, Garske-Román U (2015). Method dependence, observer variability and kidney volumes in radiation dosimetry of ^177^Lu-DOTATATE therapy in patients with neuroendocrine tumours. EJNMMI Phys.

[11] Breen SL, Publicover J, De Silva S, Pond G, Brock K, O’Sullivan B, Cummings B, Dawson L, Keller A, Kim J, Ringash J, Yu E, Hendler A, Waldron J (2007). Intraobserver and interobserver variability in GTV delineation on FDG-PET-CT images of head and neck cancers. Int J Radiat Oncol Biol Phys.

[12] Vorwerk H, Beckmann G, Bremer M, Degen M, Dietl B, Fietkau R, Gsänger T, Hermann RM, Herrmann MKA, Höller U, van Kampen M, Körber W, Maier B, Martin T, Metz M, Richter R, Siekmeyer B, Steder M, Wagner D, Hess CF, Weiss E, Christiansen H (2009). The delineation of target volumes for radiotherapy of lung cancer patients. Radiother Oncol.

[13] Foster B, Bagci U, Mansoor A, Xu Z, Mollura DJ (2014). A review on segmentation of positron emission tomography images. Comput Biol Med.

[14] King MA, Long DT, Brill AB (1991). SPECT volume quantitation: influence of spatial resolution, source size and shape, and voxel size. Med Phys.

[15] Grimes J, Celler A, Shcherbinin S, Piwowarska-Bilska H, Birkenfeld B (2012). The accuracy and reproducibility of SPECT target volumes and activities estimated using an iterative adaptive thresholding technique. Nucl Med Commun.

[16] Pacilio M, Basile C, Shcherbinin S, Caselli F, Ventroni G, Aragno D, Mango L, Santini E (2011). An innovative iterative thresholding algorithm for tumour segmentation and volumetric quantification on SPECT images: Monte Carlo-based methodology and validation. Med Phys.

[17] Fleming JS, Alaamer AS (1998). A rule based method for context sensitive threshold segmentation in SPECT using simulation. Phys Med Biol.

[18] Zaidi H (1996). Organ volume estimation using SPECT. IEEE Trans Nucl Sci.

[19] Erdi YE, Wessels BW, Loew MH, Erdi AK (1995). Threshold estimation in single photon emission computed tomography and planar imaging for clinical radioimmunotherapy. Cancer Res.

[20] Mortelmans L, Nuyts J, Van Pamel G, Van den Maegdenbergh V, De Roo M, Suetens P (1986). A new thresholding method for volume determination by SPECT. Eur J Nucl Med.

[21] Montagnat J, Delingette H (2005). 4D deformable models with temporal constraints: application to 4D cardiac image segmentation. Med Image Anal.

[22] Floreby L, Sjögreen K, Sörnmo L, Ljungberg M (1998). Deformable Fourier surfaces for volume segmentation in SPECT. Pattern Recognition, 1998. Proceedings. Fourteenth International Conference On.

[23] Floreby L, Sörnmo L, Sjögreen K (1998). Boundary finding using Fourier surfaces of increasing order. Pattern Recognition, 1998. Proceedings. Fourteenth International Conference On.

[24] Geets X, Lee JA, Bol A, Lonneux M, Grégoire V (2007). A gradient-based method for segmenting FDG-PET images: methodology and validation. Eur J Nucl Med Mol Imaging.

[25] Hatt M, Cheze le Rest C, Turzo A, Roux C, Visvikis D (2009). A fuzzy locally adaptive Bayesian segmentation approach for volume determination in PET. IEEE Trans Med Imaging.

[26] Berthon B, Marshall C, Evans M, Spezi E (2016). ATLAAS: an automatic decision tree-based learning algorithm for advanced image segmentation in positron emission tomography. Phys Med Biol.

[27] Kass M, Witkin A, Terzopoulos D (1988). Snakes: active contour models. Int J Comput Vis.

[28] Staib LH, Duncan JS (1996). Model-based deformable surface finding for medical images. IEEE Trans Med Imaging.

[29] Otsu N (1979). A threshold selection method from gray-level histograms. IEEE Trans Syst Man Cybern.

[30] Gustafsson J, Ljungberg M, Sjögreen Gleisner K (2012). Automatic segmentation of SPECT images using Fourier surfaces. Eur J Nucl Med Mol Imaging.

[31] Dice LR (1945). Measures of the amount of ecologic association between species. Ecology.

[32] Ljungberg M, Strand SE (1989). A Monte Carlo program for the simulation of scintillation camera characteristics. Comput Methods Programs Biomed.

[33] Segars WP, Bond J, Frush J, Hon S, Eckersley C, Williams CH, Feng J, Tward DJ, Ratnanather JT, Miller MI, Frush D, Samei E (2013). Population of anatomically variable 4D XCAT adult phantoms for imaging research and optimization. Med Phys.

[34] Segars WP, Sturgeon G, Mendonca S, Grimes J, Tsui BMW (2010). 4D XCAT phantom for multimodality imaging research. Med Phys.

[35] Brolin G, Gustafsson J, Ljungberg M, Sjögreen Gleisner K (2015). Pharmacokinetic digital phantoms for accuracy assessment of image-based dosimetry in ^177^Lu-DOTATATE peptide receptor radionuclide therapy. Phys Med Biol.

[36] Gonzalez RC, Woods RE (1993). Digital image processing.

[37] Press WH, Teukolsky SA, Vetterling WT, Flannery BP (1992). Numerical recipes in C: The art of scientific computing.

[38] Sjögreen Gleisner K, Brolin G, Sundlöv A, Mjekiqi E, Östlund K, Tennvall J, Larsson E (2015). Long-term retention of ^177^Lu/ ^177m^Lu-DOTATATE in patients investigated by *γ*-spectrometry and *γ*-camera imaging. J Nucl Med.

[39] Frey EC, Tsui BMW (1996). A new method for modeling the spatially-variant, object-dependent scatter response function in SPECT. Nuclear Science Symposium, 1996. Conference Record., 1996 IEEE.

[40] Garkavij M, Nickel M, Sjögreen-Gleisner K, Ljungberg M, Ohlsson T, Wingårdh K, Strand SE, Tenvall J (2010). ^177^Lu-[DOTA0,Tyr3] octreotate therapy in patients with with disseminated neuroendocrine tumours: Analysis of dosimetry with impact on future therapeutic strategy. Cancer.

[41] Sjögreen-Gleisner K, Rueckert D, Ljungberg M (2009). Registration of serial SPECT/CT images for three-dimensional dosimetry in radionuclide therapy. Phys Med Biol.

[42] BIMP, IEC, IFCC, ILAC, ISO, IUPAC, IUPAP, OIML. International vocabulary of metrology — basic and general concepts and associated terms (VIM). www.bipm.org/en/publications/guides/#vim JCGM 200:2012.

[43] Jentzen W, Weise R, Kupferschläger J, Freudenberg L, Brandau W, Bares R, Burchert W, Bockisch A (2008). Iodine-124 PET dosimetry in differentiated thyroid cancer: recovery coefficient in 2D and 3D modes for PET(/CT) systems. Eur J Nucl Med Mol Imaging.

[44] Berthon B, Marshall C, Evans M, Spezi E (2014). Evaluation of advanced automatic PET segmentation methods using nonspherical thin-wall inserts. Med Phys.

[45] Jentzen W, Freudenberg L, Eising EG, Heinze M, Brandau W, Bockisch A (2007). Segmentation of PET volumes by iterative image thresholding. J Nucl Med.

[46] Jentzen W (2010). Experimental investigation of factors affecting the absolute recovery coefficients in iodine-124 PET lesion imaging. Phys Med Biol.

[47] Koral KF, Dewaraja Y (1999). I-131 SPECT activity recovery coeffcients with implicit or triple-energy-window scatter correction. Nucl Instrum Methods Phys Res A.

